# Disentangling the complexity of mobility of older medical patients in routine practice: An ethnographic study in Denmark

**DOI:** 10.1371/journal.pone.0214271

**Published:** 2019-04-16

**Authors:** Jeanette Wassar Kirk, Ann Christine Bodilsen, Ditte Marie Sivertsen, Rasmus Skov Husted, Per Nilsen, Tine Tjørnhøj-Thomsen

**Affiliations:** 1 Clinical Research Centre, Copenhagen University Hospital Hvidovre, Hvidovre, Denmark; 2 Department of Exercise and Health, Roskilde Municipality, Roskilde, Denmark; 3 Clinical Research Centre, Physical Medicine & Rehabilitation Research-Copenhagen (PMR-C), Hvidovre, Denmark; 4 Division of Community Medicine, Department of Medical and Health Sciences, Linköping University, Linköping, Sweden; 5 Department of Health and Social Context, National Institute of Public Health, University of Southern Denmark, Copenhagen, Denmark; Plymouth State University, UNITED STATES

## Abstract

**Aim:**

Many older medical patients experience persistent functional limitations after hospitalization, such as dependency in activities of daily living, recurring fall incidents and increased mortality. Therefore, increased activity and mobilization during hospitalization are essential to prevent functional decline in older medical patients. No previous studies have explored how the social context influences how health professionals decide whether or not to mobilize patients. This qualitative study aimed to explore how social contextual circumstances affect the mobility of older medical patients in medical departments.

**Methods:**

An ethnographic field study was conducted in six medical departments in three public hospitals in the capital region of Copenhagen, Denmark. Participant observations were carried out from January to June 2017. The researchers were present for up to 14 days (range, 8–14 days) in the six departments. A total of 210 pages of field notes were produced. The participants were health professionals involved in the care of older medical patients: physiotherapists, registered nurses, nursing assistants and physicians. A content analysis was conducted.

**Findings:**

Five themes concerning mobility of patients emerged: (1) materialities; (2) professional roles; (3) encouraging moments; (4) patients and relatives; and (5) organization and management. Of these, professional roles seem to be the most important because it pervaded all themes. Different health professionals in the medical departments recognized, spoke and acted based on different cultural models.

**Conclusion:**

It was found that mobility of older medical patients is entangled in a complex network of social contextual circumstances. Mobility of older medical patients is based on health professionals’ different cultural models, which shape distinct professional identities and lead to contradictions and blurring of the priorities and responsibilities among the health professionals involved in mobilization. The consequence is that no profession “owns” the responsibility for mobilization, thus restricting mobilization of the patients during hospitalization.

## Introduction

Acute admissions and inactivity due to bed rest are independent risk factors for death and dependency in older medical patients [1,2]. It has been shown that older medical patients who lose function during hospitalization have a decreased ability to recover [3]. Accordingly, many older medical patients experience persistent functional limitations after hospitalization, such as dependency in activities of daily living, recurring fall incidents and increased mortality [3–5].

It has been shown that immobile patients in contrast to patients with high mobility levels often experience suboptimal physical and psychosocial outcomes, slower recovery, more functional decline and longer stays in hospital, and a number of well-known complications related to hospitalization such as pneumonia, deep-vein thrombosis, urinary tract infections and ulcers [6]. Therefore, increased activity and mobilization during hospitalization are essential to prevent functional decline in older medical patients.

Nurses have a key role in mobilization of older medical patients’, ensuring upright time (walking and standing) and facilitating activities that enhance the patients mobility. Mobilization and prevention of immobilization is considered a central nursing activity within several nursing diagnosis frameworks, care models and grand theories [6–8]. However, research has shown that mobilization in daily practice is not viewed as a prioritized nursing care activity by nurses [9,10]. For example, an American ethnographic study by Doherty-King et al. [11] showed that the most frequent mobility activities directed at older patients were standing and transferring from bed to chair, with a mean time per mobility event of 1.6 and 0.2 minutes, respectively. These activities were often carried out in relation to a particular medical procedure. Similarly, a Danish study by Pedersen et al. [12], which used accelerometer data, showed that older medical patients who walked independently (with or without walking aids) at admission spent a median of 17 hours a day in bed during hospitalization and walked less than 1 hour a day. These findings emphasize the importance of research on circumstances that increase mobility in hospitalized older patients.

### Barriers to mobilization

Barriers to mobilization in hospital settings have been identified at three levels: the individual, professional and organizational [9,11]. Barriers at the individual level include, for instance, doubts by nurses and physicians regarding patients’ motivation for mobilization during acute illness. They are afraid of fall injuries to patients and sustaining injuries to themselves (eg, back injuries) while mobilizing the patients [13]. At a professional level, nurses do not perceive mobilization as a part of their core tasks [14]. Thus, the professional level is related to professional identity, understood as a profession’s way of thinking, language codes, explanatory forms and routines of action and what they consider to be their core tasks [15]. Barriers at an organization level include lack of staff and time [9] and problems with teamwork and communication [14]. Also the presence of medical equipment, intravenous lines and lack of walking aids are perceived as barriers to mobilization by both physicians’ and nurses [16].

A potential organization-level barrier for physiotherapists is restricted time to examine patients and support their mobility due to changes in health care systems, which have led to expectations for a higher patient turnover [17]. The organization of physiotherapy presents another potential organizational barrier for mobility of patients [17]. In most Danish hospitals, physiotherapists belong to a central physiotherapy department, which means that the physiotherapists are not part of the permanent staff employed in the medical departments, and they therefore have limited contact with patients. Thus, physiotherapists only attend medical departments to carry out tasks related to mobility and rehabilitation of specifically referred patients.

Although studies have identified barriers to ensuring adequate mobility of older medical patients [9,11,14], no previous studies have explored how conditions of the social context influence how health professionals, individually as well as collectively, decide whether or not to mobilize patients. The present study addresses this knowledge gap in existing research by exploring social contextual circumstances of achieving mobility of older medical patients during hospitalization.

### Aim

The aim of this study was to explore how social contextual circumstances affect the mobility of older medical patients in medical departments.

According to Poland and colleagues [18], social context can be defined as “circumstances or events that form the environment within which something exists or takes place which therefore helps make phenomena intelligible and meaningful (interpreting something in context versus out of context)” [18]. Drawing inspiration from this definition, this study focuses on how materialities and social interactions influence health professionals’ perceptions and their actions regarding mobility. Materialities are physical surroundings and objects, for instance aids and technologies, that health professionals use in social interaction with colleagues and patients and which, therefore, are assumed to have importance for the health professionals’ identity, development and boundaries [19].

Ontologically, this study assumes that relationships between people and materialities affect the formation of professional identities and health professionals’ ways of acting, interacting and reacting [20].

**Fig 1:**
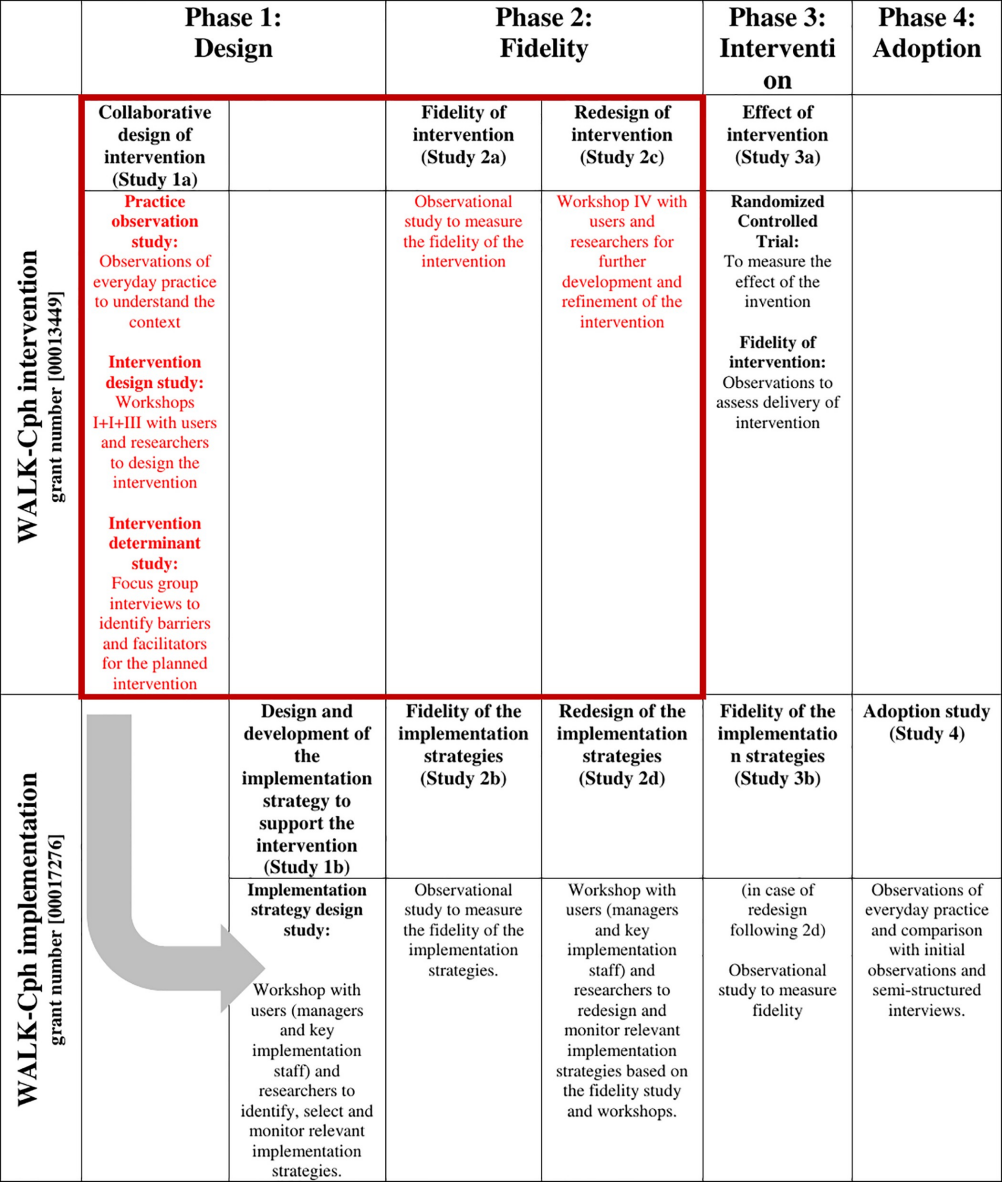
WALK-Cph intervention and implementation activities.

This study is a part of the WALK-Copenhagen project (WALK-Cph), which is designed as a mixed-methods clinical research project aimed at investigating mobility in older medical patients and evaluating an intervention to achieve increased mobility during acute hospitalization and after discharge [21]. The definition of mobility includes mobilization; many activities in and around patients in a medical department are also related to passive transfer [20]. The intervention study consists of an intervention design phase developed by means of user engagement to ensure a high degree of contextual adaptation of the intervention to facilitate implementation [22]. The design phase comprises a series of qualitative studies, an ethnographic field study, interviews and workshops (Fig 1). This study presents the main findings from the ethnographic field study.

## Methods

### Study setting

The study was carried out in Denmark, where the health care system is publicly funded from taxes. The Danish welfare state provides free treatment for primary medical care, hospitals, and home-based care services for all citizens. The ethnographic field study was conducted at six different medical departments in three public university hospitals in the capital region of Copenhagen, Denmark: (1) endocrinology; (2) infectious diseases; (3) pulmonary diseases; (4) gastroenterology; (5) general medical department; and (6) emergency department. Three of the departments were located at one hospital, two departments in another hospital and finally one department in a third hospital. The departments reflected the diversity in medical specialties, and how different medical contexts frame mobility practices in medical patients. The departments vary in size and have between 18 and 40 beds. The proportion of nurses, doctors and certified nursing assistants are almost similar in each department. In all hospitals, the physiotherapy service is centrally organized to service all wards.

### Study design

A qualitative approach was chosen to explore how social contextual circumstances and materialities frame the health care practitioners’ decisions and actions regarding mobility in older patients. We chose ethnography to explore contextual factors and culture because ethnography is the study of people in naturally occurring settings or “empirical fields” using methods of data collection that capture their ordinary activities and social meanings. We considered qualitative methods such as field study and participant observations well suited for providing in-depth contextual knowledge about the interplay between materialities and professional decision making and practice, and, therefore, crucial for an exploration of the circumstances that shape the health professionals’ reasoning and actions regarding mobility [23].

Participant observations were carried out between January 2017 and June 2017. We were present for up to 14 days (range, 8–14 days) in the six departments, ending observations in one department before moving to new department. By being physically present in the department, taking part in and observing the health professionals carrying out their daily activities, our aim was to understand how mobility of older patients is perceived and practiced in the departments by different health professionals [24].

### Data collection

The observations were carried out by four of the researchers behind this study, two of whom are registered nurses (DMS, JWK) and two are physiotherapists (ACB, RH). We used a focused observation strategy [25] whereby we followed the health professionals (physiotherapists, nurses, nursing assistants and physicians) in their daily work with a particular focus on how they talked about mobility, how they interacted with patients, and how the physical surroundings and use of different materialities such as bed lifts or walking aids shaped their actions, interactions and decisions regarding mobility of patients. Our position was primarily observant but we also asked all the health professionals that we followed on the days of observation to explain their decisions about mobility and concrete actions in specific situations. Thus, we combined observations with go-along interviews, a form of interviewing where the researcher accompanies individual informants in their natural environment; this approach enables the researcher to explore the environmental context together with participants in real time [26–28]. Members of the research team were present for between 4 and 6 hours each observation day, distributed between day and evening shifts.

We used an observation guide to ensure that the observations were systematic and to record activities, interactions and descriptive data (ie, sex, profession and professional experience) (see example in Tables 1 and 2). In the field notes, we described observations of both non-verbal and verbal aspects such as body language, dialogue between the health professionals and patients, and the use of objects such as walking aids. Dialogues were written down as close to verbatim as possible [29]. Because we were observing in combination with go-along interviews, we expanded our field notes after each observation day to provide more detail. In total, 210 pages of field notes were produced.

**Table 1:** Observation guide I.

Profession	Sex	Work experience in general	Work experience in the department	Who initiated mobilization?	What argument is used for rejection or acceptance? (motive)	What is discussed with the group or physiotherapist regarding mobilization	Are material artefacts used? Which ones? How?	Patient status
Ph**ysician**	Male	17 years	9 years	None	None	Nothing	No	Acute ill

**Table 2:** Observation guide II (researcher reflection).

Date and time	Physical room	Verbal communication	Non-verbal communication	Social consensus. How meaning is created and shared between the health professionals	Other things
**11. Jan**	The nursing room	Talking about functional decline and staying in bed		The physiotherapist and the physician disagree about the status of the patient. The physician wants to send the patient home while the therapist assesses the patient to be too weak	The physician raises his voice every time he speaks

After each observation period, we discussed and cross-checked data between DMS, JWK, ACB and RH to secure consensus about verbal and non-verbal actions to document. Also, to discuss interesting or surprising observations, which we decide to follow in the subsequent observations, to understand if they were single cases or patterns of behaviours. After conducting observations in three out of six medical departments we discussed our preliminary data with TT-T to strengthen the validity of the results [25].

### Participants

The participants were health professionals involved in the care of older medical patients and included physiotherapists, registered nurses, nursing assistants and physicians. The selection of health professionals was dependent on staffing on the days of observation, and the nursing frontline manager chose who we could follow each day. In total, we followed 61 physiotherapists, registered nurses and nursing assistants (Table 3) and 18 physicians in their rounds, conferences and board meetings.

**Table 3:** Characteristics of the health professionals observed in six medical departments.

Health profession	n (missing information)	Female gender (%)	General experience (months), median (range)
Nurse assistant	16 (4)	100	153 (2–276)
Registered nurse	33 (13)	100	27 (3–360)
Physiotherapist	12 (9)	92	64 (60–144)

### Ethical issues

Ethical approval was not required for the study because formal ethical approval is not mandatory for studies that do not involve biomedical issues according to Danish law (I-Suite no. 05078). This approval was stated by the Research Ethics Committees of the Capital Region and the Danish Data Protection Agency. The project adheres to the directives of the Helsinki Declaration [30]. Anonymity was ascertained by assigning participants a code instead of using their full names in the field notes. The researchers maintained a confidential file of identifiers tied to participant backgrounds so that the interview data (recordings and transcripts) could be coded as a basis for in-depth analysis. Before undertaking observations, all participants were informed about the aim of the study and were assured that participation was voluntary and that they and the results would be anonymized. Because the head and ward managers approved our participation in the daily practice, written informed consent was not obtained from the health professionals before the study. However, each health professional was given the opportunity to withdraw from being followed in their daily work, but none of the participants did so. During the field study, we tried to ensure that we acted with respect to the older medical patients and the health professionals by not encroaching on their private space of actions. We acted on the basis of situational ethics by combining our knowledge of the current empirical context with intuition, sense, morality and responsibility [31].

### Data analysis

The analysis began after conducting observations in the first three medical departments (endocrinology, infectious diseases and emergency department) and was carried out throughout the process of data collection in the other departments. Thus, part of the analysis progressed simultaneously with the data collection. After the fieldwork, the first author carefully read and re-read each of the 210 pages of field notes to gain an understanding of the content. Meaning units, understood as paragraphs containing aspects related to each other through their content and context, were systematically selected [32], understood as specific units of text, either a few words or a few sentences with a common meaning were grouped (see examples in Table 4). Content analysis was conducted and followed by a process to condense the meaning units whereby codes were grouped into subthemes and finally into themes [33] (Table 4). To strengthen the validity of the analysis ACB reviewed the analysis and JK and ACB discussed the results until there was consensus and for securing trustworthiness. Subsequently, we discussed and cross-checked data and interpretations with TT-T to further strengthen the validity of the results [25]. Quotations were selected from the transcripts of field notes and dialogues to illustrate the themes. Attention was paid to ensuring that these were representative of the groups’ practices and views and not just certain participants.

**Table 4:** Examples of meaning units, condensed meanings units, subthemes, and themes from content analysis of observations from the medical departments.

Meaning unit	Condensed meaning unit: description close to the text	Condensed meaning unit: interpretation of the underlying meaning	Subtheme	Theme
My first impression of the department is the corridors are very broad with high ceilings. It is an old hospital and the department is characterized by this	The department has broad corridors with high ceilings. It is an old hospital	The architecture of the department supports that there is room for mobilization and exercise There is room for mobilization and exercise which is supported by the architecture of the department	The corridor as a physical space is both a specific tool for mobilization and a sign for (the lack of) mobilization	Materialities
Two older women are walking with walkers in the corridor while talking	Two female patients are walking in the corridor with walkers	The presence of patients in the corridor becomes a symbol of mobilization taking place		
It is 11.00 a.m. and I have only observed one patient in the corridor	One patient walks in the corridor	The corridor is not a space that patients use for mobilization		
The nurse explains that it actually is a private bed room but that a relative has been sleeping in there and that she wants the other bed removed because she cannot mobilize the patient to the toilet	The extra bed has to be removed otherwise there is not room for mobilization	The amount of physical objects and size of the room has influence on whether mobilization is successful	Physical space has an influence on mobilization via design, presence or lack hereof	
There are no chairs in the bedroom because there is no room for them and the gentleman we are going to see is placed furthest away from the toilet; he can only just pass through the room with his walker. The toilet is almost the same size as the bedroom	There are no chairs in this bed room and there is only just room for a walker. The toilet is very big	There is a difference in the architecture of the ward and the design of the rooms		
The nursing assistant states that the dining room was removed many years ago and hence they have nowhere to mobilize the patients to	The removal of the dining room is the argument as to why the nursing assistant does not mobilize the patient	Changes to the architecture influence whether mobilization is supported by the staff		

## Results

Data analysis showed that mobility of patients is entangled in a complex network of contextual circumstances that can be described in terms of five main themes: (1) materialities; (2) professional roles; (3) encouraging moments; (4) patients and relatives; and (5) organization and management. The five main themes include 17 subthemes, illustrating the complexity of the issue (Table 5). The results are presented pertaining to these five themes.

**Table 5:** Themes and subthemes.

No.	Themes	Subthemes
1.	Materialities	The design of physical spaces and objects, their presence (or absence) are important for mobility
		Isolated patients
2.	Professional roles	Differences in which tasks, actions and language are considered important for mobility influence patient mobility
		Servicing self-supporting patients
3.	Encouraging moments	Certain moments enable or inhibit mobility
		Relationships on two levels
4.	Patients’ and relatives’ influence on mobility	Mobility is influenced by patient motivation
		Verbalization and practical assistance from relatives’ support mobility
		Lack of practical assistance from relatives is a barrier to mobility
5.	Organization’s and the management’s influence on mobility	Time and temporality
		Mobility is influenced by differences in medical specialities
		A safe discharge versus flow culture
		Rituals at interprofessional meetings
		Mobility is influenced by political decisions
	Other enablers and inhibitors for mobilization	Mobility has a gender perspective
		Gap between ideal and reality
		The condition (medical, mental, etc.) of the patient

### Materialities

One major theme that emerged from observations was the close connection between materialities and the mobilization of older medical patients. In particular, the size and design of patients’ rooms and access (or lack of) to, for instance, bathrooms, are important to support the patients’ mobility. The following excerpt illustrates the importance of easy access to bathrooms but also how small patient rooms and lack of space complicates mobilization:

It is much easier to get the patients out of the beds when there is space in the patient room. When it is a big patient room it is possible to move around. In the smaller patient rooms it is difficult to help patients get around, also if the patients use a walking aid. If we need to be two or more to support the patient in the small rooms, it is often easier to help the patient wash in bed rather than try to get the patient out to the bathroom. It is a good thing there are bathrooms connected to all the patient rooms because otherwise mobilization would be completely hopeless (nurse 4).

The lack of dining or living rooms was another issue that the health professionals attached great importance to when explaining why the patients stayed in the bed. Three of the six departments had both dining and living rooms, but we observed that these were rarely used. If they were used, it was often relatives who helped the patient to the dining room. When asked why, the nurses and nursing assistants conveyed that they perceived the dining rooms as uninviting with old furniture and a cold atmosphere. However, they said that they used the rooms on special occasions such as Christmas Day and New Year’s Day.

In all departments, the corridor played an important role in mobilization of patients, regardless of the presence of living and dining rooms. We heard nurses and nursing assistants telling the patients that they could use the corridor to go back and forth or to eat in. Sometimes nurses asked the patients:

Do you want to go out to the corridor and eat your food?

Often patients answered that they would rather stay in the patient room and would like to have their food served in the bed, which was frequently carried out by the nurses.

In some departments, physiotherapists used the corridor as a room for patient exercise with a rollator or high chair. However, such exercises only took place during daytime because the physiotherapists were not present in the evening.

In most of the departments, we observed that many objects such as lifts, wheelchairs and chairs were placed in the corridor. This made it difficult to navigate or walk through the corridor for both patients and health professionals. In one department, the corridor was designed as an exercise course which the patients could use for mobilization by themselves. Here, we observed that some patients and sometimes relatives went to the corridor and some patients used the exercise course. Generally, in most departments, mobilization in the corridors was rare, and when mobilization took place, it mainly happened in the patient room from bed to chair. Part of the explanation for the lack of use of corridors was that many patients refused to move out to the corridor. The reasons were ambiguous; some felt weak and others felt exposed. A physiotherapist explained:

I do understand why some patients do not want to sit in the corridor—they are too weak. They can hardly manage to get out to the bathroom (physiotherapist 2).

Mobilization was not only conditioned by space, design and access to bathrooms and dining rooms; it was also conditioned by the existence, access to and design of different forms of aids, eg, walking aids. A physiotherapist expressed:

The lack of walking aids makes it difficult to mobilize the patients. Also the design is central because it is of great important whether the walking aid has small or large wheels, and what we can obtain (physiotherapist, 4).

However, in several departments we observed that the available walking aids were not used and few patients asked for a walking aid. The nurses told the patients that it was the physiotherapist who had to explain and instruct the patients in the use of the walking aid, especially when the patient was discharged. But when we asked a physiotherapist about this issue, she explained:

It is true that it is a task for therapists to order a walking aid, but that does not mean that other health professionals cannot instruct patients in the use of the aid. But we know that they [nurses and nursing assistants—our interpretation] see it as a therapeutic task (physiotherapist 7).

To summarize, materialities such as the size of the spaces in relation to the activities that had to be carried out, design, atmosphere and accessibility, were shown to be important for the mobilization of patients. Similarly, health professionals and patients’ motivation and inclination for using special rooms eg the corridor, were also important. But the materialities were also associated with different professional identities and boundaries, which are discussed in the next section.

### Professional roles

One of the social contextual circumstances that we observed that affected mobility of older medical patients was that nurses and nursing assistants serviced self-reliant patients. Many patients asked for help getting something to drink, getting clean sheets or wanting to know when the physicians entered the department. A nurse explains:

I walk a lot of steps during the day because the patients call for more coffee, which I have to pick up in the corridor where we have placed the coffee machine for self-supporting patients and their relatives or they want me to change their sheets. It is interesting because a lot of the patients do it by themselves at home (nurse 16).

When asked why she prioritized these tasks for self-supporting patients she answered:

There are rules and procedures whereby patients should not pick up sheets by themselves in our closet. I have to pick it up for them (nurse 16).

But when the conversations were about bringing something to drink, eg, coffee in the corridor, the nurses responded:

It is more convenient to do it by myself than help the patient out to the corridor. It takes a long time and we are under time pressure already (nurse 5).

And an assistant added:

I also think it is a habit. It is so grounded in us that patients are in the hospital and they need care (assistants 4).

Our analysis shows that therapists, nurses, nursing assistants and physicians attached different meanings to concepts of mobilization and to the tasks and actions that can be considered as mobilization. In this section, we demonstrate how professional roles, identities and boundaries influenced mobilization. A nurse expressed:

Mobilization of the patients is important. We all learned that during our education, but in our daily work it is not so simple. Quite often we are very busy; therefore I do not mobilize the patient. I have to prioritize other tasks, such as medicine administration. I often make sure that the patient is mobilized to a chair. Exercise, I leave that to the physiotherapists. They are the ones who exercise (nurse 11).

Even through nurses and assistants considered mobilization important, they only mobilized the patients from bed to chair. In general, nurses made a distinction between exercise and mobilization, placing the responsibility for exercise on the physiotherapists. Nevertheless, even though the nurses talked about mobilization of the patients, we observed that mobilization was often excluded.

Physiotherapists' emphasized exercise as their core task and, like nurses and assistants, they also distinguished between exercise and mobilization:

Our daily practice consists of pulmonary physiotherapy, preparation of rehabilitation plans and … yes, exercise. Exercise is a part of the treatment. The nurses’ or the physicians’ can send a request for exercise, but when we receive a requisition for mobilization; we call the department and tell them, that mobilization is not one of our tasks (physiotherapist 3).

Although therapists emphasized exercise as a core task and a part of the treatment, we rarely observed them exercise with the patients. When we asked why, one physiotherapist answered:

Exercise is part of our core task, but pulmonary physiotherapy and rehabilitation plans come first. Then exercise (physiotherapist 8).

When the physicians focused on mobilization and exercise it was usually regarding discharge or transfer to other departments. A physician reasoned:

It is a good thing that we have therapists to make rehabilitations plans. The rehabilitation plans support me in my decision about discharge of the patients (physicians 8).

A morning meeting was held every day in the department where the physicians presented what position and responsibility they had that day. Interestingly, physicians educated in the geriatric specialties had a broader focus on mobilization. We observed that these physicians more often asked questions regarding how much the patient had been out of bed each day, and they frequently invited the physiotherapists into dialogue about the future plan for the patient and made goals for how nurses and nursing assistants could cooperate in relation to mobilization. Physicians educated in the medical specialties had a strong focus on diagnosis, treatment and discharge of the patient, which may explain why they did not have a special focus on patients’ functional abilities and mobilization.

Thus, nurses, nursing assistants and physicians talked primarily about mobilization, whereas therapists talked about exercise. Furthermore, therapists assigned particular value to exercise, pulmonary physiotherapy and rehabilitation plans and considered them as part of the treatment. Although nurses, nursing assistants and physicians considered mobilization important for the patients, they did not perceive mobilization as part of the treatment and their core tasks. Therefore, mobilization tended to fade into the background in everyday practice at the hospital. However, nurses, nurse assistants and physicians with experience from geriatric specialization prioritized mobilization to a greater extent.

### Encouraging moments

During the field work, we observed several situations where different health professionals verbally encouraged the patient to get out of the bed. Such encouraging moments were crucial for mobilization of patients. However, the actual effect of encouragements was often related to the patients’ expectations of the health professionals’ core tasks. When a physiotherapist encouraged the patient to get out of bed, it increased the chance of the patient actually leaving the bed and becoming more physically active than only sitting in a chair. Patients distinguished between the health professionals when they entered the patient’s room. As one patient explained:

When it's a physiotherapist, then you know what it's about. Then you just have to get out of bed. You know it is a physiotherapist because they are dressed so sporty (field notes, day 3).

When a nurse or nursing assistant encouraged the patient to be active, many patients rejected this advice and remained in the bed. A patient stated:

No thanks, I would like to stay in bed, but if you want to get me a cup of coffee it would be nice (field notes, day 12).

Many nurses and assistants did not follow up on the encouragement on mobilization, because mobilization was not perceived as a core task. However, again we observed that nursing assistants with experience from the geriatric specialties succeeded in mobilizing the patients. They were more insistent and did not hesitate to tell the patient to mobilize. A nursing assistant explained:

When I worked in the geriatric department, getting the patients out of the beds and mobilized was a natural part of the treatment. We all have learned that as a part of geriatric education. In this department (medical department), mobilization is not a priority (nursing assistance 8).

Nurses who did not succeed in mobilizing the patient when using encouragement used rewards instead.

I often motivate the patients by rewards, eg, I will change the sheets if the patient walks out to the corridor or finds an extra piece of cake. It helps sometimes (nurse 12).

Interestingly, patients also placed different expectations on different health professions, as illustrated in the following excerpt:

Many patients tell us that we are so nice and busy and that we have so many different things to do. Quite often they denied getting out of bed, saying that they think the physiotherapist will come and take care of their exercise (nurse 16).

The use of verbal encouragement became important in relation to professional identities and the patients' perceptions and expectations about what different professions are expected to do. Uniforms are also a symbol for a common identity, symbolizing skills and competence. The patients expected the physiotherapists who wore “sporty clothes” to be responsible for their exercise and tended to comply with this form of mobilization. The patients did not, however, assign nurses the same responsibility for mobilization, because they expected nurses to be too busy with other health-related tasks. This presumption seemed to result in patients not mobilizing when the nurses asked them to.

At rounds, the physicians asked the patients whether they had been getting out of bed, focusing on whether the patients were ready to be discharged. A nurse explains:

When the physicians ask the patient to get out of bed at rounds, some patients show the physicians that they can get of bed and be discharged (nurse 11).

The physicians seldom asked the patients just to motivate them to mobilize, with an interest in ensuring that the patient did not experience functional decline.

### Patients and relatives' influence on mobility

As already indicated, the patients' motivation for getting out of bed was crucial for achieving mobilization. Sometimes patients took the initiative and asked for assistance:

Some patients ask me if I will give them a hand getting out of bed. They want to stretch their body a little. I do understand they get so stiff from lying in bed all day (physiotherapist 5).

An important source of motivation was what we conceptualize as “transfer value”. That is when patients anticipated that mobilization in hospital would support movements and actions in their future everyday life outside hospital, eg, mobilizing on stairs because they have to be able to climb stairs before they can be discharged to their own home.

However, the overall finding was that patients did not want to get out of bed or sit in a chair. The nurse asks a patient to get up from bed to chair to eat lunch. The patient refuses and says that it must be one of the privileges of being hospitalized that you can stay in bed all day. The nurse tries to persuade the patient, but without success (field notes, day 7). A nursing assistant explained:

Many of the older patients do not want to get out of bed. It seems that they were raised to believe that when you are ill, you must recover in bed (nursing assistant 7).

Some patients also refused to receive a walking aid. We observed patients who articulated that receiving a walking aid or having to be transferred by a lift from bed to chair made them feel old or signalled unwanted dependency. One example of this was when a nurse tried to persuade an older patient to use a cane to help him mobilize to which the patient humorously responded:

Canes are only for older men. I will only be 84 years old in two weeks (field notes, day 11).

Other patients found walking aids to be of great help in their daily living, especially if the physiotherapists carefully instructed and trained the patient in the use of the aid.

An important observation was that the presence of relatives influenced whether or not mobilization of the patient was supported. When relatives asked for or demanded that the patient was mobilized during hospitalization, the health professionals gave more attention to getting the patient out of bed.

A relative complains to the physician that his mother has not been out of bed for the last two days. The physician entered the conference room and asks the nurses if it is correct that this patient has not been mobilized for the last two days. The nurses explain that they have assessed the patient to be too ill and the therapist did not have the time to make a functional assessment. The physician demands that the physiotherapist prioritize this patient and the nurses must make sure that the patient is mobilized to a chair (field notes, day 13).

Also, relatives’ resources were shown to be important. When patients were discharged, some relatives refused to receive the help offered from the municipality, even if it could increase mobilization for the patient. They used justifications like they did not want strangers in their home or they did not have the energy to focus on the patient's lack of mobility.

To summarize, both patients’ and relatives’ motivations were shown to be crucial for mobilization and especially the transfer value was an important motivational factor for the patients.

### Organization and management influence on mobility

Besides the above-mentioned contextual circumstances, time and temporality emerged from the analysis as organizational circumstances that influenced mobilization. The different professions appeared to have different rhythms of work and procedures that affected whether mobilization succeeded or not.

If only the therapists held morning meetings at the same time as us, then we could collaborate about getting the patient out of bed. I know that the therapists do not think this is their task, but it would be smart. Then they could make a functional assessment and I could clean the bed (nurse 8).

Every morning, in all departments, the nurses and nursing assistants individually planned their work day and decided which tasks they should prioritize. Interruptions that created break-ups in temporality of work such as planning, scheduling, rhythms and timing of work tasks become barriers for mobilization. A nurse expressed:

If it is just me who can plan and work as I have scheduled then it is possible for me to plan mobilization as a task I have to do….but then a physiotherapist, a student or a physician interrupts me in my work and then it all becomes a mess. Then mobilization gets a lower priority (nurse 2).

Other circumstances that interrupted the rhythm of work were missing equipment, patients having blood samples taken, acute problems with other patients or the patient ringing the bell all the time. Such break-ups become significant for the exclusion of mobilization.

Further, day and evening shifts were important. Fewer staff in the evening meant that if the patients could not stand or walk without personal aid, he or she depended on the presence of relatives to get out of bed or into the corridor. If the health professionals managed to help the patients to sit in the corridor, it could take a long time before they were able to help the patient back to bed depending on how it suited the rhythm and timing of work tasks, as shown in the following example.

It can be difficult to accommodate the patient's desire to get into the bed at a certain time. I have IV medicine to be given at a special time or the food has to be served. Then the other patients have to wait in the corridor (nurse 16).

On an organization level, different rhythms of work and different types of interruptions that created break-ups in the temporality of work affected whether mobilization succeeded or not.

## Discussion

This study sought to explore how social contextual circumstances affected mobility of older medical patients in six medical departments in Denmark. Bed rest, low mobility and low physical activity have been problems for years and are still mentioned as targets for intervention by politicians, health professionals and researchers [34,35]. The present study shows that mobility of patients is entangled in a complex network of social contextual circumstances within five interrelated themes that emerged from the content analysis: materialities; professional roles; encouraging moments; patients and relatives; and organization and management. In the following we first discuss the themes separately. We end the discussion by identifying and considering the one theme, professional roles, that we consider to be the most important, because it pervades all themes.

A central finding of the study was the close connection between materialities and the mobility of older medical patients, which is in line with other studies [9,36]. For instance, an American study by Brown et al. [9], focusing on barriers to mobility during hospitalization, emphasized the importance of lack of chairs or televisions in the corridors. In addition, our study points to the importance of physical spaces for mobilization, both in terms of size and accessibility, but also for the patients’ and health professionals’ motivation for using these spaces. A Danish qualitative study about home and homeliness in Denmark [37] explored how spaces may become a room where the objects are associated with social activities. Gregory [38] argues that space both shapes and is shaped by human activity. In our study, it appeared that some spaces were not used and therefore not shaped by human activities. Even though half of the departments had dining or living rooms, they were seldom used by nurses or nursing assistants to mobilize the patients. The nurses and nursing assistants considered these spaces uninviting, with old furniture and a cold atmosphere. Therefore, these spaces were seldom transformed into rooms. They were only used on special occasions, such as at Christmas and New Year, when they were transformed from uninviting spaces into inviting rooms associated with social community. On these occasions, the health professionals made an extra effort to mobilize the patients to these rooms.

Other studies have identified corridors as a physical space where patients mobilize themselves or sometimes with assistance from nurses or physiotherapists [11,36]. However, in our study, the use of corridors for mobilization was uncommon. An American study by Drolett et al. [39], with the aim of determining the effectiveness of a nurse-driven mobility protocol to increase the percentage of patient mobility during the first 72 hours of their hospital stay, showed that physiotherapists were only available to exercise the patient 30 minutes per day. In Denmark, patients only receive physiotherapy if it is prescribed by a physician. In our study, few patients exercised with physiotherapists because the physicians rarely prescribed exercise and the physiotherapists tended to prioritize pulmonary physiotherapy and rehabilitation plans (administrative work) over exercise. Furthermore, in many departments, the corridor was used to store aids and other objects. According to Winter [37], people recognize rooms and the use of them with reference to the objects that are located in the rooms. A corridor that looks like a storage room does not invite mobilization or exercise. Only one department has designed their corridor as an exercise or mobilization course, with arrows on the floor and posters showing different types of exercises, which seemed to motivate the patients and relatives to go to the corridor for exercise. Thus, this design transformed the corridor into an exercise room, and the arrows and other objects mediated this transformation. The use of arrows and posters altered patients’ behaviour in a predictable way [39]. This finding suggests that the architecture and design of built spaces can motivate social activities and are therefore crucial for mobilization.

The importance of aids in relation to mobility is connected to their availability and design, as mentioned in other studies [2,9]. However, our study also indicates that materialities are important in other ways. Our results show that materialities are an inevitable part of the creation of social identities, underscoring that materialities and people are entangled with each other and one cannot be understood without the other [19]. For instance, patients accepted to receive an aid to facilitate mobility if they perceived the utility of this aid in supporting and helping them in their ordinary life. However, if patients did not believe the aids matched their self-perception in terms of, for example, age or dependency, they declined to use the aids.

Similar to other studies, we found that patients’ lack of motivation was an important factor for mobilization [9,14]. Some patients felt too ill to move or considered it a privilege to stay in bed all day when hospitalized. Interestingly, the transfer value of exercising or mobilizing emerged as a special motivational factor for patients to mobilize. Thus, patients’ motivation for mobilization increased if they realized that exercises and mobilization could help them in their daily life after discharge. This finding is consistent with a Dutch study by De Vreede et al. [40], which concluded that exercises that are consistent with everyday life make them easier to sustain, and that patients were less motivated to perform exercises that were less transferable to their daily life situations.

However, we also found that patients’ motivation depended on the profession of the person who initiated mobilization. In line with the study of Brown et al. [9], the patients in our study did not assign the same responsibility for mobilization to nurses as, for example, to physiotherapists. When nurses and assistants used rewards such as cakes or clean bedsheets as motivational incentives, it seemed to reinforce the patients' perception of the nurses and nursing assistants’ responsibility to undertake tasks other than mobilization. In contrast, when the physiotherapist entered the patient’s room and verbally encouraged the patient to get out of bed, this was consistent with the patients’ expectations concerning the professional identity of the physiotherapist. For the patient, getting out of bed was interpreted as meaningful in the social context of the physiotherapist and the patient, which increased the success with getting patients out of bed.

Several studies have identified lack of time as a barrier to mobilize or exercise [9,14,36], but we found that time availability depended on the way the health professionals were organized; interruptions to their plan and rhythm of work were important for mobilization of older medical patients. The physiotherapists only worked during the day, limiting their opportunity to examine patients. The consequence of this organization was that mobilization or exercise in the evening depended on the nurses or nursing assistants on evening duty, who were often busy doing other tasks. Many of the nurses and nursing assistants talked about not having the time to mobilize the patients. Sorokin and Merton [41] have described the social dimension of time, arguing that our experience of time reflects cultural patterns that give time meaning. Even though nurses or nursing assistants individually planned their day in the morning, this planning reflects a collective and cultural pattern about how to act and which tasks they had to prioritize individually and collectively. Nurses and assistants were often interrupted during the day, which led to re-planning and re-prioritizing of their tasks. This meant that mobilization was often deprioritized by other tasks such as medication administration. The physiotherapists, nurses and nursing assistants also worked within different temporal rhythms, which became a challenge for the mobilization of the patients. Thus, when talking about time as a contextual circumstance of importance for mobilization, we found that it was not only objective time such as clock time or lack of time that hindered mobilization [42] but also different profession-conditioned temporal rhythms at a collective level, reflecting boundaries between different professions and their collective understandings of how to work and prioritize in a meaningful manner.

Professional roles, identities and boundaries seemed to pervade all the themes. A qualitative study by Doherty-King and Bowers [14] identified two groups of nurses: those who claimed that mobilization of patients was their responsibility and those who attributed the responsibility to other professions. All nurses and nursing assistants in our study talked about having a responsibility to mobilize the patients, but we found that, if they mobilized the patients, it was often only from the bed to sit in a chair. On the one hand, the level of mobilization could be interpreted as a discrepancy between rhetoric about having the responsibility for mobilization (“espoused theory”) and what was actually observed in daily practice for nurses and nursing assistants (“theory in use”) [43]. This is in line with other studies that have shown that nurses most often engage patients in low-level activity (standing and transferring in connection with care tasks) [5,14,36]. On the other hand, the result could also be interpreted as mobilizing from bed to chair was the level of mobilization that made sense for nurses and nursing assistants in relation to their professional identity. A literature review by Kalish et al. [44] of current research evidence concerning the outcomes of mobilizing hospitalized adults concluded that mobilization was the most reported lack among nursing tasks and that mobilization was considered a problem for the nurses and often omitted [14,35,44].

In the study by Doherty-King and Bowers [14], in which nurses took responsibility for mobilization, interprofessional collaborations increased. The argument about the benefits of interprofessional collaboration has been invoked internationally for many years [45] with the argument that when individuals from different professions learn together they will work better together, improving care and the delivery of service [46]. This argument has a strong appeal for working in the context of significant organizational and attitudinal barriers, encouraging them to create interprofessional learning opportunities. Despite interprofessional learning, it has been shown that embedding interprofessional learning into health profession curriculum remains a challenge, including logistic and cultural factors. Especially, cultural challenges between interprofessional collaborations in daily practice and in education are a challenges [47]. Lack of supportive health service environment and concerns from senior staff that mono disciplinary learning may be comprised of barriers for interprofessional learning. Hereby the influence of the hidden curriculum [48] and prevailing cultural norms become significant. However, we found that nurses, nursing assistants and physicians trained in geriatrics often succeeded in mobilizing patients. One explanation could be that the geriatric specialty historically has had a focus that is not limited to a single diagnosis and profession, but rather on older patients with multiple concurrent diseases and changes in age and function [49]. These foci are integrated parts of the geriatric specialty, thus shaping the professional identity of health professionals trained in this specialty. However, further research is needed to understand these social contextual circumstances.

### The pervasiveness of professional roles and identities

In the final part of the discussion, we consider the pervasiveness of professional roles and identities and how it affects mobilization of older medical patients. We take inspiration from anthropologist Cathrine Hasse’s concepts of cultural models [50], which she defines as a collection of knowledge that creates connections between objects, actions and what is important about these subjects. These connections create simplified worlds that yield expectations about how each health profession should act, react, talk, use objects and appear both internally in the profession but also between different professions. When physiotherapists in our study, in contrast to nurses, nursing assistants and physicians, talked about exercise, pulmonary physiotherapy and rehabilitation plans and wore sporty clothing, they collectively constructed their professional identity with reference to what they consider as their core tasks and what other professions can expect them to do. When they use words such as exercise instead of mobilization, the language becomes a social product, which emphasizes their professional identity. The physiotherapists perceptions of the concept of mobilization is related to care tasks and nursing and therefore not related to their professional identity. Thus, physiotherapists talk and act with a background in a cultural model that defines their professional identity, thereby creating boundaries in relation to mobilization.

Nurses and nursing assistants used the word mobilization rather than exercise as part of their professional identity and their cultural model. However, most often, it was an espoused theory rather than a theory of use because to the nurses and nursing assistants, mobilization could be included or excluded in their daily work, thereby becoming a part of a negotiation and a selection process in which they define whether mobilization was important for their professional identity. Quite often it was not perceived as important for their professional identity and was excluded. These negotiations were not only important within their own profession; they also took place between different professions. Mobilization could thus be interpreted as a power game concerning the distribution of tasks and responsibilities between different professions [51]; a game that was particularly evident in the relationship between the physiotherapists and the nurses. Physiotherapists did not see mobilization as a task related to their professional identity but as a nursing task; instead they considered older medical patients’ rehabilitation plans as a core task [17]. Therefore, many physiotherapists in our study focused on supporting mobility by evaluating the patient’s dependency and the potential need for aids to support independent basic mobility. Another example of boundaries linked to differences in cultural models of professional identities between the nurses and the physiotherapists was expectations and responsibilities for how materialities affected mobilization. Physiotherapists had the responsibility for ordering, eg, the walking aid, but the responsibility for instructing the patients in the use of the aids was not unambiguous. Thus, nurses expected that it was the physiotherapists who instructed the patients in the use of the aids, whereas the therapist did not perceive this task as obvious for them and expected the nursed to take care of this task. The consequence was that patients sometimes were discharged without instruction on the aids.

The physicians were physically absent more often in our study, because their tasks in other departments left limited time for visits to the department. In contrast to Doherty-King et al. [14], where physicians had a legal obligation to order activity for mobility and daily prescriptions of activity, physicians in Denmark are only legally required to order rehabilitation if the patient has experienced a functional decline. Across the hospitals and the six departments the physicians did not consider mobilization to be a core task for them in their daily work. They seldom spoke about who had the responsibility for getting the patients out of bed. Rather, their focus was on diagnosis and treatment, and they only addressed mobilization when it helped them in decisions about whether or not a patient could be discharged.

### Implications of the findings

The findings from the study raise several questions. If we increase the number and availability of walking aids and other materialities, will it then be possible to increase patients’ mobility? Or if we increase the staff resources and thereby provide more time, is it then possible to increase the patients’ mobility during hospitalization? Our study shows that mobility of older medical patients not only depends on single determinants, such as the number of chairs or availability of resources, but that mobility of older medical patients is a social practice where nurses, nursing assistants, physiotherapists and physicians recognize, speak, act and interact based on different cultural models, blurring the responsibility for mobilization of older medical patients. The consequence is that no profession “owns” the responsibility for mobilization in daily practice, and the patients are not mobilized during hospitalization. This is supported by the study of Pedersen et al. [12], showing that older medical patients spend a median of 17 hours a day in bed during hospitalization and walk less than 1 hour a day. Therefore, future research and interventions need to address and investigate not only barriers at an individual level from nurses, physicians or patients [9,11,14,36] but also mobilization as a collective social practice that depends on relationships and collaborations at organizational, professional and individual levels, and accordingly all the health professionals involved in the mobility of older medical patients.

The results of this study may help health professionals to focus their attention on the social contextual circumstances that affect mobilization of patients and work with awareness and explicitly about how they, in collaboration among different professions, patients and relatives, can motivate patients to stay out of bed. The physicians could, for instance, use their authority during rounds as an opportunity to encourage and motivate the patients and their relatives. The nurses and nursing assistants might be able to increase their awareness about the duality between mobilizing the patients as a part of their professional role or service the patients as a part of the care-giving role, which is also deeply embedded in their professional identity, and because it is easier and faster for them to do it by themselves in daily practice. In addition, the results can be used for all professions to reflect on how the concepts of exercise in contrast to mobilization becomes a discourse barrier of relevance for the mobility of older medical patients.

### Strengths and limitations

This study has both strengths and limitations that should be considered when interpreting the findings. By involving researchers with different professional backgrounds, we ascertained a broad spectrum of relevant health care professionals in daily contact with older medical patients in need of mobilization and work-related tasks of mobilizing older medical patients.

As a guiding principle for reporting the study, we have use the Standards for Reporting Qualitative Research (SRQR) [52], which we believe strengthens the validity and the transparency of the ethnographic field study.

We acknowledge that the ability to gather data and generate knowledge depends on the position of the researcher [25], a position that we did not always define ourselves but were assigned by the health professionals when we followed them in daily practice. By comparing, cross-checking and discussing our observations, we became aware of how our different professional backgrounds and perspectives framed our observations and data generation. Based on this procedure we learned how our professions created different observation foci. E.g. physiotherapists observed how the interaction between them and the patients created special expectations regarding mobility which led to time out of bed. Whereas, the observing nurses noted how care was provided as service. These observations helped us to be aware of interprofessional differences and to collect and interpret data with background in our former observations on a more common position. We believe this has strengthened the validity of the results [53].

We found that being present in the departments for up to 14 days was sufficient for us to take an accepted position in relation to the health professionals and to distinguish between the typical and the unique regarding mobilization situations [23]. Credibility was enhanced by systematizing the observations and creating transparency [54] and this enabled us to explore and understand how health professionals make decisions about mobility in interaction with the patients and each other. Transferability of the findings was supported through descriptions of the time and context in which the data were obtained.

We ensured the validity of the findings by comparing results along the way and searching for inconsistent findings. According to Davies [55], validity depends on the “ethnographic truth” gained through immersion in field work. We had the opportunity to return to the same department and visit new departments, to compare statements with observations and to observe whether the topic was relevant in different contexts [55]. We believe that the trustworthiness of the findings was enhanced through systematic discussions between JWK, ACB and TTT, followed by discussions among the research team to ensure codes, subthemes and themes were adequately described. Credibility was established through use of field notes and verbatim statements to illustrate the findings, and themes were later presented to health professionals, patients and relatives during workshops [25].

One limitation was that the physicians were only present in the departments for a short time. We observed that they were not particularly focused on mobility of older medical patients. Whether or not they discussed mobilization in other areas outside the department is not known. In a future study, the ethnographic field study will be followed by semi-structured interviews with physicians to get a deeper insight into whether mobility of older medical patients is a focus that concerns the physicians.

## Conclusions

The present study found that mobility of older medical patients is entangled in a complex network of social contextual circumstances and not only depends on single determinants, such as access or lack of space, number of chairs or availability of human and time resources, but that mobility of older medical patients is a social practice where the different health professionals working in medical departments recognize, speak and act based on different cultural models. These cultural models, shaping distinct professional identities, lead to contradictions and blur the priorities and responsibilities among the health professionals involved in mobilization of older medical patients, with the consequence that no profession “owns” the responsibility for mobilization in daily practice, and the patients are not mobilized during hospitalization.
